# Chondrocalcinosis does not affect functional outcome and prosthesis survival in patients after total or unicompartmental knee arthroplasty: a systematic review

**DOI:** 10.1007/s00167-021-06519-6

**Published:** 2021-03-06

**Authors:** Céline S. Moret, Edna Iordache, Riccardo D’Ambrosi, Michael T. Hirschmann

**Affiliations:** 1 Department of Orthopedic Surgery and Traumatology, Kantonsspital Baselland, 4101 Bruderholz, Switzerland; 2 IRCCS Istituto Ortopedico Galeazzi, Via Galeazzi 4, 20161 Milan, Italy

**Keywords:** Chondrocalcinosis, Unicompartmental knee arthroplasty, Total knee arthroplasty, Calcium pyrophosphate dihydrate, Outcome, Implant survival

## Abstract

**Purpose:**

There are contentious data about the role calcium pyrophosphate (CPP) crystals and chondrocalcinosis (CC) play in the progression of osteoarthritis (OA), as well as in the outcomes after knee arthroplasty. Hence, the purpose of this systematic review was to analyse the clinical and functional outcome, progression of OA and prosthesis survivorship after unicompartmental knee arthroplasty (UKA) and total knee arthroplasty (TKA) in patients with CC compared to patients without CC.

**Methods:**

A systematic review of the literature in PubMed, Medline, Embase and Web of Science was performed using the “Preferred Reporting Items for Systematic Reviews and Meta-Analysis” (PRISMA) guidelines. Articles which reported the outcome and survival rates of prosthesis after TKA or UKA in patients with CC were included.

**Results:**

A total of 3718 patient knees were included in eight selected publications, with a median sample sizes of 234 knees (range 78–1000) and 954 knees (range 408–1500) for publications including UKA and TKA, respectively. At time of surgery, the mean age was 69 years and the prevalence for CC ranged from 12.6 to 36%. Chondrocalcinosis did not significantly influence the functional and clinical outcome, the implant survival as well as the radiologic progression of OA disease after UKA and TKA.

**Conclusion:**

The presence of CPP crystals in tissue samples, synovial fluid or evidence of calcifications on preoperative radiographs did not significantly influence the postoperative functional and activity scores. It also had no significant influence on prosthesis survival rate, whether it was a UKA or a TKA. This study shows that the impact of a subclinical form of chondrocalcinosis may not be of clinical relevance in the context of arthroplasty.

**Level of evidence:**

IV.

## Introduction

Chondrocalcinosis (CC) refers to calcifications of hyaline cartilage and/or fibrocartilage, detected by imaging and/or histological examination, most frequently caused by the formation of calcium pyrophosphate (CPP) crystals in the pericellular matrix of the chondrocytes as seen in calcium pyrophosphate deposition (CPPD) disease [1, 2].

There is a strong association of chondrocalcinosis with age [3, 4]. The prevalence in the adult population varies from 3.7% in those aged 55–59 years to 17.5% in those aged 80–84 years [4].

In patients with end-stage osteoarthritis (OA), the prevalence of histological and/or radiological chondrocalcinosis varies from 30% up to 53% [5, 6]. There is an association between chondrocalcinosis and OA and both are common in the elderly [7–10].

On the one hand, chondrocyte apoptosis, associated with age, contributes not only to cartilage matrix degradation and OA but also to an increase in substrate production required for the formation of CPP crystals [11–13]. Hence, the chondrocytes present in the OA knee are more prone to generate CPP crystals and thus to cause chondrocalcinosis.

On the other hand, CPP crystals induce inflammation by activation of the NLRP3 inflammasome and through the production of metalloproteinase and prostaglandins [14, 15]. Their direct catabolic effect on chondrocytes and on synoviocytes further damages the cartilage leading to progression of OA [16–18].

In unicompartmental arthroplasty (UKA) or when a patellar resurfacing is not done in total knee arthroplasty (TKA), the remaining presence of cartilage might trigger the production of CPP crystals leading to an acute inflammation, possibly resulting in inferior functional outcome or lower prosthesis survival rate. Thus, Kozinn and Scott [19] gave the recommendation not to implant UKA in patients with chondrocalcinosis.

It was the purpose of this systematic review to analyse the outcome, progression of OA and prosthesis survivorship after UKA and TKA in patients with CC compared to patients without CC. The hypothesis was that there is no significant effect on outcomes, progression of OA or survival rates.

## Materials and methods

### Search strategy

In the electronic databases PubMed, Medline, Embase and Web of Science, a systematic search was performed from their inception until August 15, 2020 to identify relevant articles. Search terms included all synonyms for UKA and TKA as well as those for CPPD disease and chondrocalcinosis. Only articles written in English and German were found and taken in consideration. Following compilation of all identified articles and removal of duplicates, two reviewers independently assessed the studies for inclusion criteria by title and abstract. Selected articles were then scanned by full text on their eligibility. Furthermore, manual screening of references of the selected studies was performed.

All peer-reviewed articles, prospective trials and retrospective studies were considered. This review was conducted in accordance with the established guidelines of Preferred Reporting Items for Systematic Reviews and Meta-Analysis (PRISMA).

**Fig. 1:**
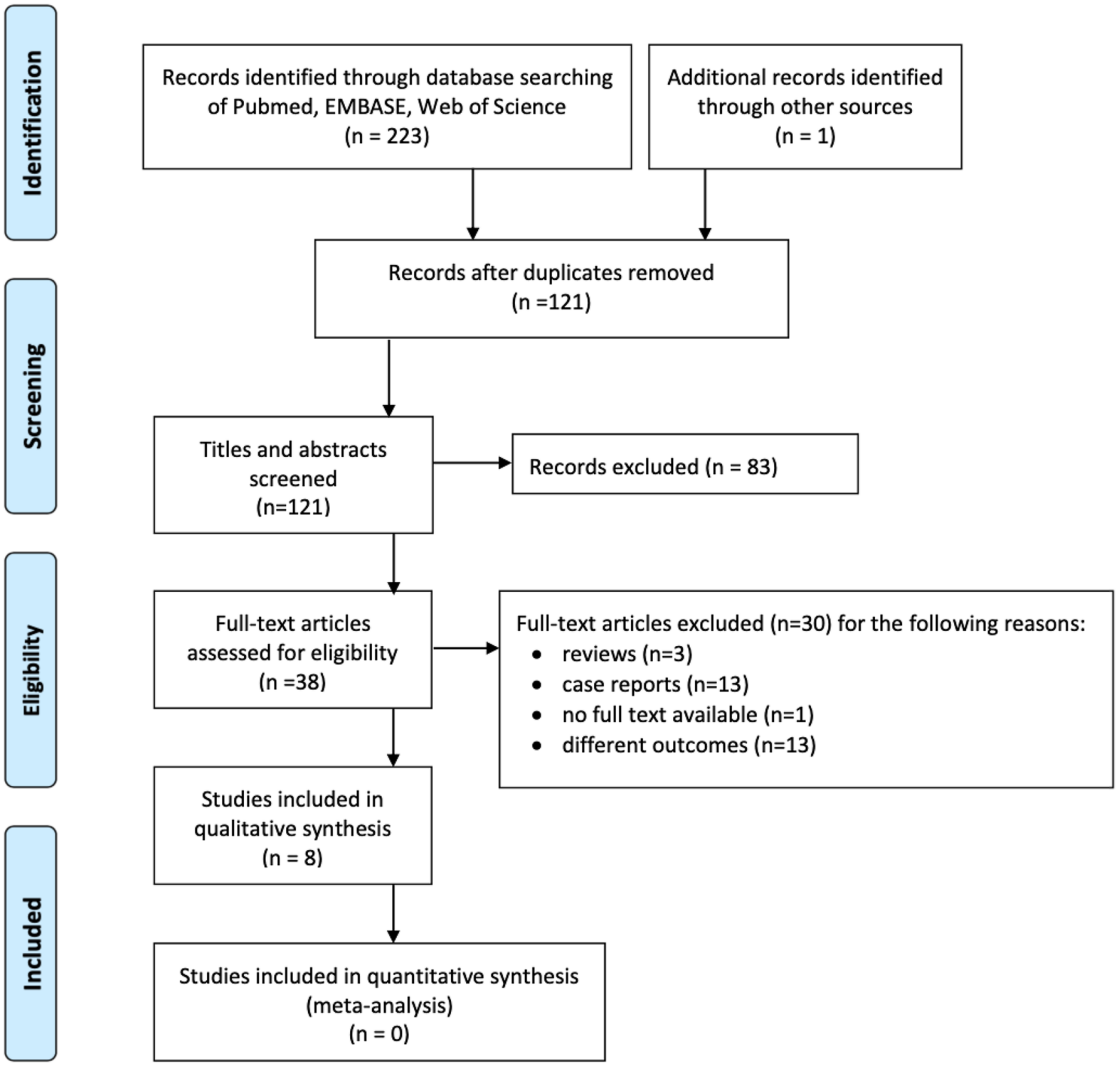
Prisma flow diagram showing the study selection process

The original studies considered for this review included outcome and follow-up of patients who underwent UKA or TKA and presented with chondrocalcinosis either preoperatively or intraoperatively. Figure 1 shows a study flow diagram with all exclusion criteria.

### Quality assessment

The methodological quality of the included studies and the risk of bias were assessed using “The Methodological Index for Non-Randomized Studies” (MINORS) for non-randomized comparative and non-comparative clinical intervention studies [20]. Each of the studies fulfilled the requirement of the MINORS appraisal tool which provides for a global ideal score of 16 for non-comparative studies and of 24 for comparative studies.

### Statistical analysis

Continuous variables were described using means and standard deviations or medians and ranges. Categorical variables were tabulated with absolute and relative frequencies. For data interpretation, a *p* < 0.05 was considered statistically significant.

Due to the large heterogeneity and the lack of randomized controlled studies, performing a meta-analysis was not possible. Moreover, since a part of the single measures distributions was available only as range and not as standard deviation (SD), no other statistical analysis was possible.

## Results

### Search results

The literature search yielded a total of 223 publications and, after allocation processes shown in Fig. 1, eight studies met the criteria for this systematic review.

### Study characteristics and quality of included studies

All included studies analysed the impact or the correlation of CC with the functional outcome, the survivorship and the progression of OA especially in UKA, but also in TKA without patellar resurfacing. There were five (62.5%) prospective cohort studies [21–25] and three retrospective cohort studies [26, 27], of which one was with prospective data collection [28]. According to the “MINORS” appraisal tool for non-randomized control trials, the mean global score was 19.5 for comparative studies.

A total of 3718 patient knees were assessed, with median sample sizes of 234 (range 78–1000) and 954 (range 408–1500) for publications including UKA and TKA, respectively. Further characteristics of the included studies are listed in Table 1.

**Table 1:** Characteristics of included studies

Authors	Woods (1995)	Pandit (2011)	Hernigou (2012)	Pandit (2016)	Hamilton (2017)	Kumar (2017)	Lee (2014)	Willems (2019)
Country	UK	UK	F	UK	UK	UK	USA	NL
Level of evidence	IV	IV	II	III	IV	III	III	III
Type of arthroplasty	Mobile bearing UKA	Mobile bearing UKA	Fixed bearing UKA	Mobile bearing UKA	Mobile bearing UKA	Mobile bearing UKA	TKA	TKA
Number of knees	98	1000	234	78	1000	369	1500	408
Female Pt. (%)	n/a	52	51.45	47.1	52	41	64.6	66
Age at surgery—mean (range or SD) y	69^a^ (50–79)	66 (32–88)	70^b^ (60–89)	68.8^a^ (48–81) (8.3)	66 (32–88)	69.8 (8.7)	70 (34–100)	68.4 (9.5)
Follow-up time—mean (range or SD) y	3.5^a^ (0.33–8)	6.4 (2.9) (min. 10 for 86 Pt.)	10 (3.4) (min. 10 for 112 Pt.)	n/a	10.3 (5.3–16.6) (min. 10 for 516 Pt.)	10 (2.9)	4.75 (2–10)	5 (4.75–7)
Prevalence of CC at surgery (%)	20.4	12.6	36 (63^c^)	n/a	13	15.2	26.4	15.4
Diagnostic methods	Histology	Radiology and/or histology	Radiology and SF analysis	Histology	Radiology and/or histology	Radiology and histology	Visual	Radiology

*Pt.* patients, *SD* standard deviation, *y* years, *CC* chondrocalcinosis, *UK* United Kingdom, *F* France, *USA* United States of America, *NL* The Netherlands, *UKA* unicompartemental arthroplasty, *TKA* total knee arthroplasty, *n/a* not available, *min*. minimum, *SF* synovial fluid

^a^Only case group

^b^Median

^c^Including patients that developed CC during follow-up

### Diagnostic methods and prevalence

In all six publications [21–25, 28] about UKA, chondrocalcinosis was considered to be present if there was either radiologically visible calcifications within the soft tissues or the cartilaginous structures preoperatively or CPP crystals seen histologically in tissue sample or with compensated polarized light microscopy in synovial fluid. In one study [28], the presence of CC was additionally determined on postoperative radiographs. Whereas Lee et al. [26] based their diagnostic criteria on the sole intraoperative, visual presence of CC and Willem et al. [27] on the radiologically visible calcifications.

Besides one publication, all [21–23, 25–28] reported a prevalence of CC at time of surgery between 12.6% and 36%. Lee et al. [26] calculated a prevalence of 26.5% based on the intraoperative, visual presence of calcium deposition.

### Outcome and survival rates after UKA

According to four studies [21, 23, 25, 28] stratifying the clinical and functional outcome by the presence of chondrocalcinosis after UKA, no significant difference was found.

These four studies established no significant differences with regard to the mean Oxford Knee Score (OKS), Knee Society Score (KSS), Tegner Activity Scale (TAS) and pain scores, as well as to the self-reported performances of daily activities at last follow-up [21, 23, 25, 28]. However, patients with CC showed a significantly worse TAS preoperatively and the difference in the OKS from preoperative to postoperative (delta OKS), was significantly higher in patients with CC, especially in those with histologic CC but not in those with sole radiological CC findings [21, 22]. Table 2 shows the clinical and functional outcomes.

**Table 2:** Clinical and functional outcomes (scores) at last follow-up

Authors	Woods (1995)		Pandit (2011)		Hernigou (2012)		Pandit (2016)		Hamilton (2017)		Kumar (2017)			Lee (2014)		Willems (2019)		
Group	CC	N-CC	CC	N-CC	CC	N-CC	OARev	N-Rev	CC	N-CC	1 2 3	CC H-CC R-CC	N-CC N-CC N-CC	CC	N-CC	CC	N-CC	
OKS (mean, SD)	n/a				n/a		n/a				1	43 (7)	41 (8)	n/a				
											n.s							
			42.4 (7.0)	40.9 (7.8)					39.9 (10)	39.7 (8)	2	44 (6)	41 (8)			11.4 (8.9)	14.3 (10.0)	
			*p* = 0.04						*p* = 0.17		n.s					n.s		
											3	42 (8)	41 (8)					
											n.s							
Delta OKS	n/a		n/a		n/a		n/a		n/a		1	19 (10)	15 (10)	n/a		n/a		
											*p* < 0.01							
											2	21(9)	15 (10)					
											*p* < 0.01							
											3	18 (10)	15 (10)					
											n.s							
KSS (mean, SD or range)	n/a		n/a		174 (85–196)	178 (110– 200)	n/a		n/a		n/a			n/a		n/a		
					*p* = 0.3													
KSS-KS	n/a		87.1 (11.)	84.5 (11.5)	n/a		n/a		79.8 (15)	80.4 (15)	n/a			93 (95% CI, 87–97)	94 (95% CI, 87–95)	21.7 (22.3)		18.7 (26.0)
			*p* = 0.32						*p* = 0.67					*p* = 0.52		n.s		
KSS-KF	n/a		83.0 (17.7)	83.5 (20.1)	n/a		n/a		73.7 (23)	76.4 (22)	n/a			68 (57–81)	68 (59–75)	14.5 (37.4)		15.8 (33.0)
			*p* = 0.79						*p* = 0.34					*p* = 0.60		n.s		
TAS (mean, SD)	n/a		2.9 (1.3)	2.8 (1.2)	n/a		n/a		2.5 (1)	2.5 (1)	n/a			n/a		n/a		
			*p* = 0.6						*p* = 0.16									
ROM (mean, 95% CI) °	n/a		n/a		n/a		n/a		n/a		n/a			113 (95–117)	112 ( 89–119)	n/a		
														*p* = 0.034				
Pain (%)			n/a		n/a		n/a		n/a		n/a			n/a		n/a		
None to mild at rest	95	91																
	n.s																	
Moderate to severe at rest	5	9																
	n.s																	
None to mild walking	95	88																
	n.s																	
Moderate to severe walking	5	12																
	n.s																	
AODL			n/a				n/a		n/a		n/a			n/a		n/a		
Cooking	n/a				*p* = 0.25													
Rising					*p* = 0.77													
Using restrooms					*p* = 0.88													
Going upstairs					*p* = 0.41													
Going shopping					*p* = 0.36													
AFI (mean, SD)	n/a		n/a		n/a		n/a		n/a		n/a			n/a		− 6.2 (5.1)		− 6.7 (4.9)
																n.s		

*OKS* Oxford Knee Score, *KSS* Knee Society Score, *KSS-KS* Knee Society Score-knee score, *KSS-KF* Knee Society Score-knee function, *TAS* Tegner Activity Scale, *ROM* range of motion, *AODL* activities of daily living, *AFI* algofunctional index, *SD* standard deviation, CI confidence interval, *CC* chondrocalcinosis, *N-CC* no chondrocalcinosis, OARev revision surgery for osteoarthritis progression, *N-Rev* no revision for osteoarthritis progression, *H-CC* histological chondrocalcinosis, *R-CC* radiological chondrocalcinosis, *n/a* not available, *n.s*. not significant, *HR* hazard ratio

Knees with CC did not show any significant radiological progression of OA in the contralateral compartment during follow-up [25, 28]. The frequency of aseptic loosening, mechanism of failure, revision rate and time to revision as well as the cumulative implant survival rate at 15 years was identical in both groups (Table 3) [21, 23, 25, 28]. Compared to the controls, Kumar et al. [22] found a similar 10-year implant survival rate in patients with radiologic chondrocalcinosis but slightly inferior in patients with histological chondrocalcinosis.

**Table 3:** Prosthesis survivorship

Authors	Woods (1995)		Pandit (2011)		Hernigou (2012)		Pandit (2016)		Hamilton (2017)		Kumar (2017)			Lee (2014)		Willems (2019)	
Group	CC	N-CC	CC	N-CC	CC	N-CC	OARev	N-Rev	CC	N-CC	1 2 3	CC H-CC R-CC	N-CC N-CC N-CC	CC	N-CC	CC	N-CC
Revision rate at last FU (%)	5	10	3.2	2.9	n/a		n/a		n/a		n/a			3.6	2.2	2	5
	n.s		*p* = 0.84											*p* = 0.2		n.s	
10 year survival rate (%) (95% CI)	n/a				n/a		n/a		n/a		1	91.8 (82.6–96.2)	98.3 (94.3–99.5)	n/a		n/a	
											HR 3.3 (1.0–11.7)						
											n.s						
			*96.4 *(89.0–100.0)	95.4 (91.4–99.4)							2	86.1 (69.6–94.0	99.2 (94.7–99.9)				
											HR 5.8 (1.2–28.3)						
			*p* = 0.99								*p* = 0.03						
											3	96.3 (92.4–99.6)	98.2 (92.4–99.9)				
											HR 2.9 (0.5–18.1)						
											n.s						
15 year survival rate (%) (95% CI)	n/a		n/a		87	90	n/a		91.6 (76.9–100)	92.3 (86.3–98.4)	n/a			n/a		n/a	
					*p* = 0.64				*p* = 0.75								
Time to revision surgery—mean (range) m	13 (7–19)	71 (24–91)	24 (9.6–67.2)	40.8 (2.4–120)	n/a		n/a		n/a		n/a			50 (31–98)	46 (28–113)	n/a	
	n.s		n.s		p = 0.12									p = 0.68			

*FU* follow-up, *CI* confidence interval, *m* months, *CC* chondrocalcinosis, *N-CC* no chondrocalcinosis, *OARev* revision surgery for osteoarthritis progression, N-Rev no revision for osteoarthritis progression, *H-CC* histological chondrocalcinosis, *R-CC* radiological chondrocalcinosis, *n.s*. not significant, *n/a* not available, *HR* hazard ratio

### Outcome and survival rate after TKA

After TKA, functional scores and pain improved equally, and no difference in range of motion or KSS between patients with and without CC could be demonstrated (Table 2) [26, 27]. Furthermore, no significant difference in secondary patellofemoral resurfacing or total revision rates could be observed (Table 3).

## Discussion

The major findings of this systematic review showed that chondrocalcinosis does not significantly influence the functional and clinical outcome, the implant survival as well as the radiologic progression of OA in the other compartments after UKA and TKA.

### UKA

Chondrocalcinosis was considered to be a contraindication for UKA because of its inflammatory component leading to faster OA progression and earlier revision [19].

Nonetheless, the 15-year cumulative survival rate is approximately 90% in two of the included studies [21, 28]. This is even slightly superior to the 76% to 85% found in the meta-analysis by Evans et al. [29] for the overall survival rates of UKA. With mean follow-up times ranging from 3.5 to 10 years in the other three included studies, the number of failed UKAs is even smaller [22, 23, 25]. These findings rather contradict a correlation between CC and revision rates. However, it would require a sample size calculation to estimate in a first step the number of failures which would allow to further conclude to such a correlation.

Concerning the slight inferior implant survival rate in patients with histologically proven CC compared to radiologically diagnosed CC found by Kumar et al. [22], one could argue that the preoperative diagnosis of CC, except for patients with clinical manifestation of inflammation, is usually established based on radiographic findings and not on histology, which questions the clinical relevance of this finding. On the other hand, the presence of CPP crystals either in the synovial fluid or in the cartilage, not yet visible on plane radiographs could be diagnosed non-invasively especially with ultrasound, which has a better sensitivity compared to plain radiographs, or with MRI or with dual-energy CT [30–34].

Hernigou et al. [28] collected the synovial fluid at the time of surgery and detected CPP crystals in 85 out of 234 (36%) knees. Of those, 68 (80%) had radiological signs of CC preoperatively. The rest (17/85—20%) showed subsequent signs on postoperative radiographs during follow-up. When reading all radiographs during follow-up, 63 (27%) additional knees presented with radiographic evidence of chondrocalcinosis, raising the prevalence of chondrocalcinosis at last follow-up to 63%.

This could suggest that in the early and acute form of the disease, the CPP crystals are mostly present in the synovial fluid, calcification may not have taken place yet and thus chondrocalcinosis is not visible on plain radiographs. When calcifications are solely seen on plain radiographs, one could argue that it is not possible to differentiate between different kinds of crystals. It is known that other crystal forms exist, like basic calcium phosphate (BCP) crystals, which can also be of clinical relevance and have not been analysed in the included studies [35].

Regrettably, four included studies [21–23, 25] did not report how many additional patients showed signs of CC on follow-up radiographs or whether patients with solely histologically proven CC at time of surgery subsequently presented with signs of CC on follow-up radiographs. Thus, it is impossible to determine whether the increase in CC cases during follow-up, as described by Hernigou et al.’s [28], is an isolated occurrence.

This present study demonstrates the great heterogeneity of diagnostic methods used in the included publications and that therefore analysis of the results can be open to interpretation. According to the official guidelines of the European League against Rheumatism, synovial fluid analysis should be performed using compensated polarised light microscopy since histological analysis is simply not justifiable in a native knee [2]. Radiographs can give additional information, yet its sensitivity varies depending on the population and joint between 29 and 93% [2].

The preoperative condition of the lateral compartment appears to be a significant predictor of OA progression contrary to other variables like CC and leg alignment [24]. Concerning CC, the findings of the study by Pandit et al. [24] show that only one of 26 patients presented histological signs of crystal deposition in the case group and none in the controls. Therefore, the study seems to be underpowered. Furthermore, it is declared as prospective, although biopsies were only performed on 64 out of 2,333 (2.7%) knees. Nonetheless, biopsies were performed on 24 of 26 patient in the case group.

Concerning alignment, there is contradictory evidence between Hernigou et al. [28] and Pandit et al. [24]. The former could not demonstrate a progression of OA in the contralateral compartment despite the presence of CC, which they attributed to the slight under-correction of the varus or valgus deformity they had deliberately aimed for, to decrease wear of the contralateral compartment. In their study population, revision for OA progression only occurred in overcorrected knees as supported by another of their publications [36]. Pandit et al. [24] for their part found no difference in the progression of OA in the other compartment independently of the postoperative axis of the leg, although the angle was less than 10° of valgus, which they considered to be a normal alignment based on the study by Gulati et al. [37]. However, the difference in measurement methods (radiological measurement versus clinical measurement with long arm goniometer) used by the authors [24, 37] does not allow an accurate comparison of their findings.

### TKA

In TKA, when comparing patients with different grades of CC, Lee et al. [26] observed a reduced postoperative knee flexion and KSS in patients with an intraoperative macroscopically detected high-grade CC. Yet, neither radiographs, histology, nor synovial fluid analysis was performed. The worse outcome of these patients could have been be biased by the fact that all of them underwent radical synovectomy. However, a meta-analysis reviewing five RCTs [38] about concomitant synovectomy during TKA detected no significant difference in clinical and functional KSS or range of motion postoperatively.

If paterellar resurfacing should be undertaken in TKA, especially in patients with inflammatory arthritis, remains controversial [39–42]. Reviews on the subject often exclude these patients, so that only studies with a small sample size are available [43, 44]. In one study reviewed, secondary patellar re-surfacement was performed in only 1% of patients after TKA, but not in relation to CC [27].

A meta-analysis by Evan et al. [29] mentioned a 15-year pooled survival rate of 93% (95% CI 92.8–93.1) to 96.3% (95% CI 9.7–96.9) for TKA. Therefore, the mean follow-up time of about 5 years for the included publications about TKA is too short to determine the effect of CC on long-term survival rates.

The limitations of this study were due to the small number of publications on the topic as well as their heterogeneity in regard of their patient selection, measured outcomes and different detection methods of CC. The selected study analysed between 1 and 413 knees which presented with CC at time of surgery. With such a small and heterogenous sample size, there is the risk of selection bias. In addition, these small patient samples are prone to be underpowered.

It is possible that the patients considered in the studies we have reviewed were diagnosed with CPPD disease based solely on radiological or histological findings and that they were mostly not even aware of this disease or at least not severely affected and did not present with clinical symptoms that would have required the treatment or follow-ups by a rheumatologist. Hence, these results cannot be extrapolated for patients who suffer from a symptomatic form of CPPD disease.

## Conclusion

In conclusion, this study suggests that the prevalence of chondrocalcinosis is underestimated, but that an association between OA and chondrocalcinosis exists. The presence of CPP crystals in tissue samples, synovial fluid or calcifications on radiographs preoperatively did not impact the postoperative functional and activity scores and had no significant repercussion on the survival rate of the prosthesis, being a UKA or a TKA. The pathophysiology of CPPD disease as well as its implication in inflammatory processes has been numerously investigated; nonetheless, this study shows that the impact of a subclinical form of chondrocalcinosis may simply not be of clinical relevance in the context of arthroplasty.

## Abbreviations

CC: Chondrocalcinosis
CPP: Calcium pyrophosphate deposition
CPPD: Calcium pyrophosphate deposition disease
OA: Osteoarthritis
UKA: Unicompartmental knee arthroplasty
TKA: Total knee arthroplasty
MINORS: Methodological Index for Non-Randomized Studies
SD: Standard deviation
CI: Confidence interval
HR: Hazard ratio
OKS: Oxford Knee Score
KSS: Knee Society Score
KSS-KS: Knee Society Score-knee score
KSS-KF: Knee Society Score-knee function
TAS: Tegner Activity Scale
ROM: Range of motion
AODL: Activities of daily living
AFI: Algofunctional Index
SF: Synovial fluid
Pt.: Patients
FU: Follow-up
min.: Minimum
y: Years
m: Months
n/a: Not available
n.s.: Not significant
H-CC: Histological chondrocalcinosis
R-CC: Radiological chondrocalcinosis
N-CC: No chondrocalcinosis
OARev: Revision surgery for osteoarthritis progression
N-Rev: No revision for osteoarthritis progression
UK: United Kingdom
F: France
USA: United States of America
NL: The Netherlands
